# The inventory as a core element in the further development of the science curriculum in the Mannheim Reformed Curriculum of Medicine

**DOI:** 10.3205/zma001099

**Published:** 2017-05-15

**Authors:** Julia Eckel, Katrin Schüttpelz-Brauns, Thomas Miethke, Alexandra Rolletschek, Harald M. Fritz

**Affiliations:** 1 University Medicine Mannheim, Medical Faculty Mannheim at Heidelberg University, Department of Undergraduate Education and Educational Development, Mannheim, Germany; 2 University Medicine Mannheim, Institute of Medical Microbiology and Hygiene, Mannheim, Germany

**Keywords:** Curriculum inventory, longitudinal science curriculum, training of scientific skills/competences

## Abstract

**Introduction:** The German Council of Science and Humanities as well as a number of medical professional associations support the strengthening of scientific competences by developing longitudinal curricula for teaching scientific competences in the undergraduate medical education. The National Competence Based Catalogue of Learning Objectives for Undergraduate Medical Education (NKLM) has also defined medical scientific skills as learning objectives in addition to the role of the scholar. The development of the Mannheim science curriculum started with a systematic inventory of the teaching of scientific competences in the Mannheim Reformed Curriculum of Medicine (MaReCuM).

**Methods: **The inventory is based on the analysis of module profiles, teaching materials, surveys among experts, and verbatims from memory. Furthermore, science learning objectives were defined and prioritized, thus enabling the contents of the various courses to be assigned to the top three learning objectives.

**Results:** The learning objectives systematic collection of information regarding the current state of research, critical assessment of scientific information and data sources, as well as presentation and discussion of the results of scientific studies are facilitated by various teaching courses from the first to the fifth year of undergraduate training. The review reveals a longitudinal science curriculum that has emerged implicitly. Future efforts must aim at eliminating redundancies and closing gaps; in addition, courses must be more closely aligned with each other, regarding both their contents and their timing, by means of a central coordination unit.

**Conclusion: **The teaching of scientific thinking and working is a central component in the MaReCuM. The inventory and prioritization of science learning objectives form the basis for a structured ongoing development of the curriculum. An essential aspect here is the establishment of a central project team responsible for the planning, coordination, and review of these measures.

## Zusammenfassung

**Zielsetzung: **Die Stärkung wissenschaftlicher Kompetenzen, u.a. durch die Entwicklung von longitudinalen Curricula zur Vermittlung von wissenschaftlichen Kompetenzen im Medizinstudium, wird vom Wissenschaftsrat und von verschiedenen Fachgesellschaften gefordert. Im Nationalen Kompetenzbasierten Lernzielkatalog Medizin (NKLM) wurden, neben dem Gelehrten, medizinisch-wissenschaftliche Fertigkeiten als Lernziele definiert. Auf dem Weg zum Mannheimer Wissenschaftscurriculum wurde zunächst das Ziel einer systematischen Bestandsaufnahme der Lehre wissenschaftlicher Kompetenzen im MaReCuM (Mannheimer Reformiertes Curriculum für Medizin) verfolgt.

**Methodik: **Die Bestandsaufnahme basierte auf der Analyse von Modulsteckbriefen, Lehrmaterialien, Expertenbefragungen und Gedächtnisprotokollen. Weiterhin wurden wissenschaftsorientierte Lernziele definiert und priorisiert, so dass die Inhalte der verschiedenen Lehrveranstaltungen den drei am höchsten priorisierten Lernzielen zugeordnet werden konnten.

**Ergebnisse:** Die Lernziele der systematischen Gewinnung von Informationen zum Stand der Forschung, kritischen Bewertung von wissenschaftlichen Informationen und Quellen, der Präsentation und Diskussion von Ergebnissen wissenschaftlicher Untersuchungen werden vom 1. bis 5. Studienjahr in verschiedenen Lehrveranstaltungen vermittelt. Es lässt sich ein longitudinales Wissenschaftscurriculum feststellen, welches implizit entstanden ist. In Zukunft müssen Redundanzen beseitigt und Lücken geschlossen sowie die Veranstaltungen inhaltlich und zeitlich mit Hilfe einer zentralen Koordination abgestimmt werden.

**Schlussfolgerung: **Die Lehre des wissenschaftlichen Denkens und Arbeitens ist wesentlicher Bestandteil im MaReCuM. Die Bestandsaufnahme und die Priorisierung wissenschaftsorientierter Lernziele stellen die Basis für eine strukturierte Weiterentwicklung des Curriculums dar. Essentiell ist, dass eine zentrale Steuerungsgruppe diese Maßnahmen plant, koordiniert und überprüft.

## Introduction

The strengthening of scientific competences during the undergraduate medical education is an aspect supported amongst others by the German Council of Science and Humanities [1], [2], [3], [4], [5], [6], [7]. “Scientific competences include knowledge, skills, and attitudes that are necessary for understanding, assessing, applying, and documenting scientific concepts, methods, and findings and for an active involvement in the medical learning process and the assurance of its quality” [1]. Besides the role of the scholar, the National Competence Based Catalogue of Learning Objectives for Undergraduate Medical Education (NKLM) has also defined medical scientific skills as learning objectives [7]. In order to manage the vast volumes of information [8] in the medical field, the training of generic scientific skills is required for the critical assessment of new developments in diagnosis and therapy. Fundamental methodical and scientific competences constitute a precondition for the application of evidence-based medicine (EBM) to ensure an optimum degree of patient care. These form the basis for the prompt and comprehensive integration of scientific knowledge into everyday medical care. 

A number of reviews on the teaching of medical scientific competences for students in the clinical phase of their undergraduate training and for resident physicians have meanwhile been published [9], [10], [11], presenting for example learning objectives, teaching methods, and evaluations of individual teaching courses or lecture series, e.g. for evidence-based medicine [12], [13]. Single courses for teaching scientific competences should be presented together in an integrated longitudinal coordinated curriculum. 

The so-called “Research Skill Development Framework” [14] defines a variety of competence levels in relationship to various facets of research (e.g. the retrieval or communication of scientific information) and the degree of the student’s autonomy (e.g. with guidance or on his/her own). One concept for the development of curricula that has proven its worth is the model proposed by Kern et al. (2009) [15]. This model distinguishes six separate steps:

1. problem identification and general needs assessment,
2. targeted needs assessment,
3. target the curriculum by setting goals and objectives,
4. selection of educational strategies,
5. implementation of the curriculum, and
6. evaluation.

The further development of the longitudinal science curriculum at the Medical Faculty Mannheim at Heidelberg University thus started with a systematic and comprehensive inventory [16], [17], [18], [19] of the teaching of scientific competences and skills (needs assessment) and the definition of science learning objectives (setting goals and objectives). These project stages are presented in the following paper. 

## Project description

**Figure 1:**
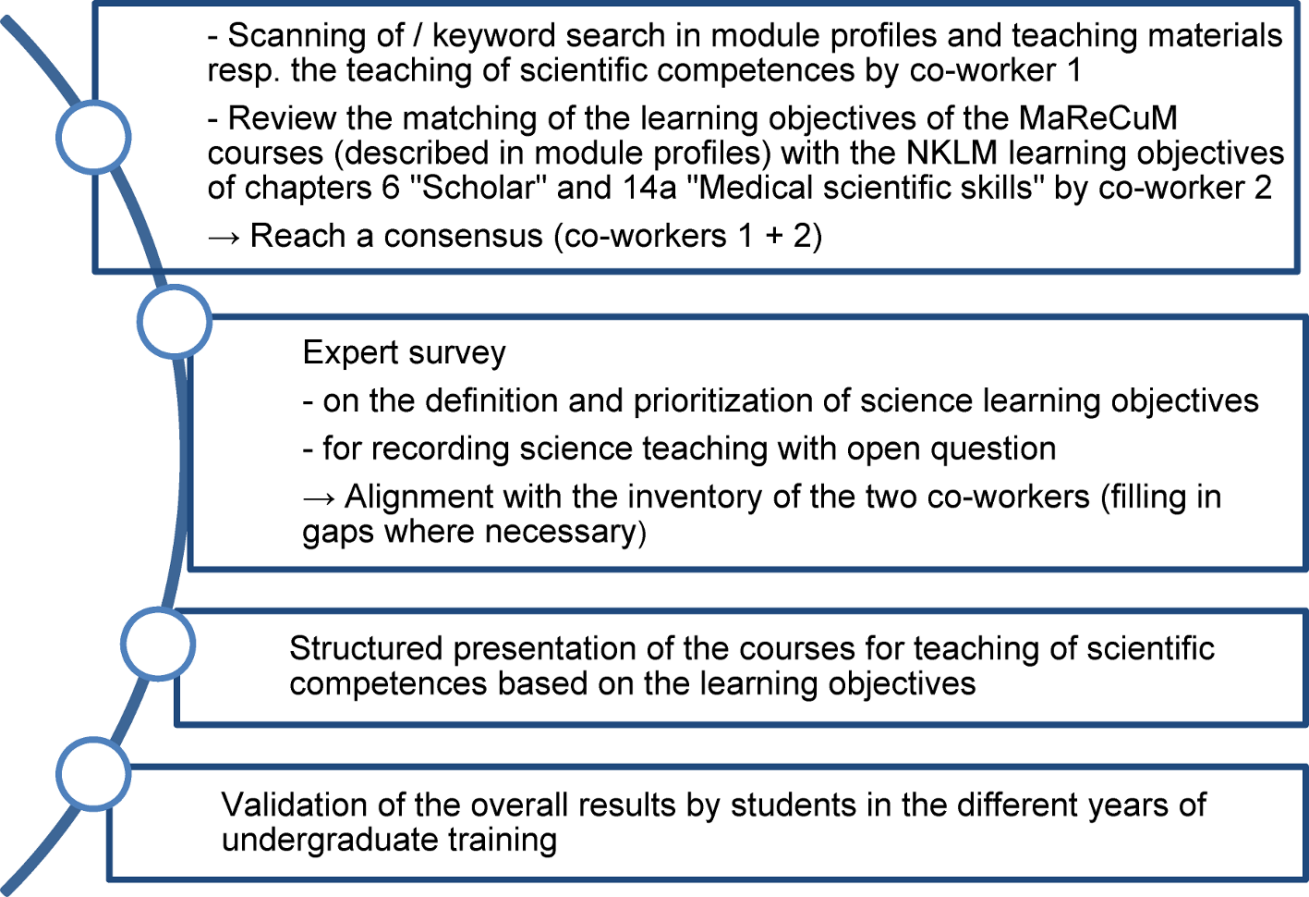
Project schedule

The first step was to set up a task force titled “Wissenschaftsstrang” (Scientific Curriculum), involving basic researchers, clinicians, medical training experts, and students’ representatives. This task force defined the specific project stages, as presented in Figure 1 (Fig. 1) and described in the following: First, the precondition for the systematic development of a longitudinal science curriculum was established in the first half of 2014 by identifying and systematically recording all courses for the teaching of scientific competences in the Mannheim Reformed Curriculum of Medicine (MaReCuM). Two project co-workers independently documented teaching courses in which scientific competences were taught. Two different methods were used to record the courses: one project co-worker diagonally scanned module profiles and teaching materials that had been placed at the students’ disposal on the MaReCuM learning management system. She also ran a search routine through these materials, using keywords such as “science”, “research”, “studies”. Another member of the project team of the MERLIN working group [20] reviewed the match between learning objectives from module profiles of the courses of the MaReCuM programme with the NKLM learning objectives defined in chapters 6 and 14a [7]. The two co-workers then compared their results. 

**Figure 2:**
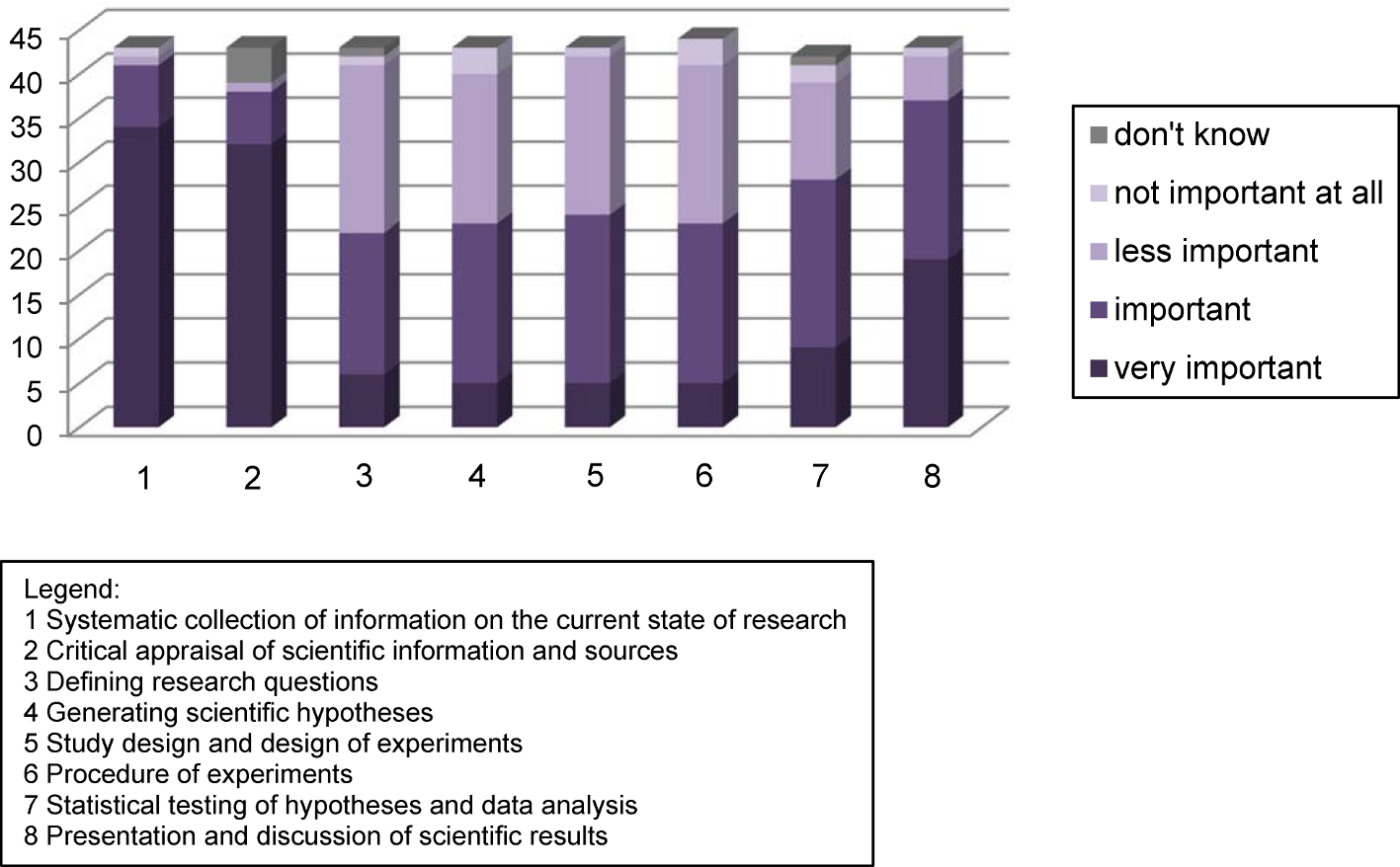
Prioritized MaReCuM learning objectives regarding medical scientific competences

After that an online survey was conducted: All medical directors, module coordinators, and members of the faculty that were responsible for single subjects in the MaReCuM programme (N=75) were asked to answer the following questions: Where and which scientific competences are taught in MaReCuM (open question)? Which scientific competences are important for medical graduates at the start of their professional career, regardless of the working environment (closed question)? Figure 2 (Fig. 2) shows the possible answers (boxes below the figure). These were developed in accordance with the NKLM, chapter 14a “Medical scientific skills”. The closed questions were evaluated descriptively (percentage of agreement to the answer options), while the open question was evaluated using a qualitative content-analysis method [21]. The evaluation of the open question of the expert survey was used to validate these answers and, wherever necessary, to add missing courses. 

This evaluation process was followed by mapping a structure of the inventory on the basis of the learning objectives as prioritized by the experts (see Figure 2 (Fig. 2)). The course contents from the different study years were assigned to the three learning objectives given the highest priority. The use of hyperlinks enabled both a compressed and a detailed presentation on the contents of the teaching courses at various levels of abstraction. At the end of the process, the final inventory was re-reviewed with one student representative from each of the five study years.

## Results

43 persons took part in the online survey (response rate = 57%). 95% of the participants stated that they were engaged in research, either in natural science, theoretical clinical research, or the practical clinical research (5% of the participants were not engaged in research).

Figure 2 (Fig. 2) shows that the learning objectives

- (1) Systematic collection of information on the current state of research,
- (2) Critical appraisal of scientific information and sources, and
- (8) Presentation and discussion of scientific results

were rated as being “very important” or “important”.

Both project co-workers arrived at almost the same results regarding the inventory. The discrepancies were then discussed and consensus was reached on the inventory. The results of the expert survey reflect the consensus reached on the results. The students in the various study years also confirmed the results of the inventory. The analyses of the open question showed that there was a lack of teacher training for special teaching methods in the science curriculum.

**Figure 3:**
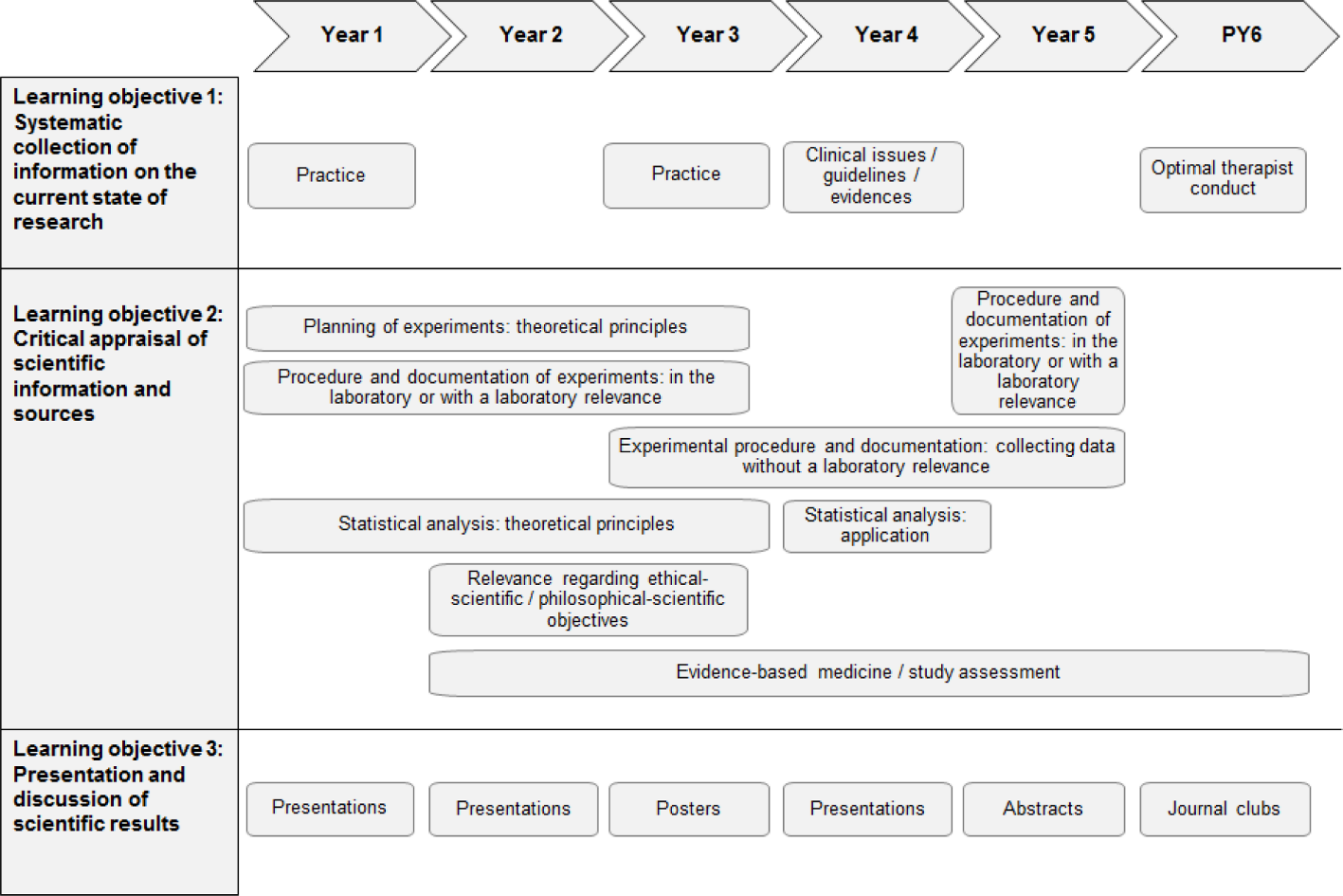
Overview of current courses for teaching scientific thinking and working in the MaReCuM programme

Figure 3 (Fig. 3) shows the results of the inventory of the science curriculum. In general, there is an implicit longitudinal science curriculum that can be identified, one that has evolved from self-interests of faculty staff in different medical fields. Overall, the inventory shows that all learning objectives of the NKLM chapters 6 and 14a have been thematized in the MaReCuM programme. The science learning objectives prioritized by the staff responsible for the undergraduate training are longitudinally addressed in the MaReCuM programme over all five years. Regarding learning objective (1), i.e. the systematic collection of information on the current state of research, it is apparent that instruction in research training is given by library co-workers in the first and third years of study. In the clinical study phase, each student is required to carry out independent literature searches for papers, case presentations, and journal clubs (whereas in the preclinical phase the materials for the presentation papers are supplied to the students). Regarding the achievement of learning objective (2), i.e. the critical appraisal of scientific information and sources, in the preclinical phase the theoretical principles for the design of experiments and statistics are taught in the courses “Biomathematics”, “Medical Psychology”, and “Ethics”, with deeper immersion into the contents following in various courses during the clinical study phase. Students are taught how to perform, record, and evaluate laboratory experiments in the basic courses in the preclinical phase. In a number of courses in the clinical phase, students are taught in the collection and evaluation of patient data using standardized measurement instruments, e.g. in the context of a pain study in the module “Primary patient care”. 

The aspects of EBM and the analysis of studies are thematized in a variety of courses spread over the whole programme of medical undergraduate training. In this regard, for example, parts of studies or complete studies are dealt with during the courses, or the applicability of guidelines is critically discussed on the basis of specific patient cases. Learning objective (3), the presentation and discussion of scientific results, is addressed in the preclinical phase in various courses on pathobiochemistry in the second and third study year, and also in a course in the module “Injuries, degenerative diseases, rehabilitation” in the fourth study year in the form of a presentation of a research paper by each student. The preparation and review of a poster presentation takes place in the module “Society and health” in the third study year. Students have to present an abstract on a paper in the module “Primary patient care” in the fifth study year. 

## Discussion

The teaching of the principles of scientific thinking and working in the undergraduate training of medicine is recommended by the German Council of Science and Humanities as well as by a number of medical professional associations. As we have been able to show, the teaching of scientific skills and competences is already an essential part of the MaReCuM. 

The MaReCuM teachers in the survey prioritized the following learning objectives: “Systematic collection of information on the current state of research”, “Critical appraisal of scientific information and sources”, and “Presentation and discussion of scientific results”. These learning objectives are also emphasized in the international context [22]. The NKLM [7] and various international frameworks [23], [24] emphasize the importance of generic skills and competences regarding the information retrieval and the critical discussion of ethics, statistics, and study designs in the scientific context. The learning objective “Dissemination of research results” is prioritized in the NKLM and in two other frameworks [23], [24]. AMEE Guide No. 69, “Developing research skills in medical students”, for example, specifies the search for and the application and communication of evidence-based knowledge as learning objectives [25]. Furthermore, a recent survey conducted among students of medicine suggests that especially the learning objective regarding the critical appraisal of scientific information and data sources should be facilitated during the course of undergraduate training: only 28% of the students think that their undergraduate training prepared them sufficiently for interpreting research results, although precisely this is one of the competences they expect to acquire [26]. 

The inventory has succeeded in revealing an implicit (naturally emerging) science curriculum that can now be further developed to an integrated longitudinal curriculum. The next steps must be to select teaching methods and to implement and evaluate training programmes [15]. The teaching courses should be aligned with each other both in terms of their contents and their timing, redundances should be eliminated, and gaps in the curriculum should be closed. In the clinical subjects, for example, basic principles regarding clinical guidelines (e.g. guidelines levels of evidence) are taught over and over. These basic concepts are going to be taught one time in the beginning of the clinical phase. Specific guidelines will be thematized in the individual subjects in the subsequent courses. We are going to use well-established teaching methods for facilitating evidence-based medicine [12], [27], [28]. The current reformation of the clinical phase in undergraduate training – MaReCuMplus – opens the opportunity to adapt the existing courses. 

As recommended by the Research Skill Development Framework [14] the learning objectives of the longitudinal scientific curriculum should be interdependent and be based on each other, like in a learning spiral [29]. Students should apply scientific principles of medicine more frequently in the clinical context. At the same time, we are pursuing the aim of integrating these learning objectives, e.g. research aspects and scientific working techniques and evidences, into already existing courses wherever possible [3]. In this regard, the “Science Curriculum,” task force has recommended to particularly emphasise the following NKLM learning objectives (chapter 14a): definition of research questions, generation of strong hypotheses according to the current state of knowledge (14a.2.1.5), the interpretation and presentation of results of statistical hypothesis testing (14a.3.1.2), and the critical discussion of the significance of scientific results from the methodological perspective (14a.3.1.3). The constructive alignment, i.e. the coordination of learning objectives, teaching methods and assessment, plays a central role [30], [31], [32]. 

In the winter term 2015/16, a new certificated module named “Principles of Scientific Method” concluding with a graded research project was introduced. The research project can be started at any time of the course of studies but must be submitted at the latest four months before the second state examination. Eligible are experimental or non-experimental research projects from the entire spectrum of medicine, including the basic sciences. As a measure to prepare students for their research project, the courses “Critical assessment of scientific evidence”, “Good Scientific Practice: Procedure in the case of misconduct”, “Scientific writing”, “Applying for grants and animal testing”, and “Clinical trials, Approval of the Ethical Review Board, Data protection, Patent-protection law” have been integrated in the curriculum of the third study year. 

MaReCuM teachers provided a toolbox with recommendations and tips on the issue of science teaching to improve the structured teaching of scientific competences and to enhance the diversity of teaching methods [33]. 

The working group is going to enhance the visibility of the science curriculum, to define implementation and communication strategies, to coordinate the teaching activities, to provide resources, and to review the efficacy of the science curriculum. 

The quality of the structure, procedure, and results of science teaching including the newly introduced “Principles of Scientific Method” module with an obligatory research project will be monitored on the basis of systematic evaluations. The levels of the Research Skill Development Framework will be applied for measuring the (changes in the) scientific competence [14].

## Limitations

The experts addressed in the online survey were all members of the MaReCuM teaching staff. It cannot be excluded that other faculties would have set different priorities regarding the learning objectives. In addition, almost all respondents pursue research activities besides their clinical occupation. For this reason, it remains unanswered if clinicians involved solely in patient care may have prioritized the learning objectives differently.

The inventory was recorded using written teaching materials and on the basis of available module profiles. There was no actual review of the science teaching in real life. Hence we cannot exclude the possibility that essential teaching contents that are not documented are missing from the inventory. However, the alignment with the contents stated by the teaching staff in the course of the survey and the review of the details provided by the students of all study years shows that we have succeeded in recording all major issues. 

## Conclusions

The structured inventory and prioritization of science learning objectives form the basis of the further development of the curriculum: thus it was possible to reveal and communicate an implicit curriculum. The development of the curriculum, however, constitutes a comprehensive and constantly ongoing process that requires the cooperation of many different faculty members. Change management processes must be initiated. A broad variety of measures must be considered in the development of structured longitudinal science curricula. It is essential that a central project coordination team is established to take responsibility for planning, coordinating and reviewing these measures. 

## Acknowledgement

We want to thank Dr Elisabeth Narciss for taking the inventory of the science curriculum. Special thanks to the students of the faculty who reviewed the final inventory. 

## Competing interests

The authors declare that they have no competing interests. 

## References

[1] Wissenschaftsrat (2014). Empfehlungen zur Weiterentwicklung des Medizinstudiums in Deutschland auf Grundlage einer Bestandsaufnahme der humanmedizinischen Modellstudiengänge.

[2] Arbeitsgemeinschaft der Wissenschaftlichen Medizinischen Fachgesellschaften e.V. (AWMF) (2015). Stellungnahme der AWMF zum "Masterplan Medizinstudium 2020".

[3] Arbeitsgemeinschaft der Wissenschaftlichen Medizinischen Fachgesellschaften e. V. (AWMF) (2014). Stellungnahme zur Wissenschaftlichkeit des Medizinstudiums.

[4] Arbeitsgemeinschaft der Wissenschaftlichen Medizinischen Fachgesellschaften e. V. (AWMF) (2008). Stellungnahme zur Förderung der wissenschaftlichen Medizin schon in der studentischen Ausbildung.

[5] Bundesvertretung der Medizinstudierenden in Deutschland e. V. (bvmd) (2014). Konzeptpapier zur Zukunft und Weiterentwicklung des Medizinstudiums.

[6] DFG Senatskommission (2010). Empfehlungen der Senatskommission für Klinische Forschung. Strukturierung der wissenschaftlichen Ausbildung für Medizinerinnen und Mediziner.

[7] Projektgruppe NKLM (2015). Nationaler Kompetenzbasierter Lernzielkatalog (NKLM).

[8] Sönnichsen A, Rinnerberger A (2008). Medizinische Informationsflut und Wissenstransfer in die Praxis – eine Quadratur des Kreises?. Schwsche Ärztez.

[9] Green ML (1999). Graduate medical education training in clinical epidemiology, critical appraisal, and evidence-based medicine: a critical review of curricula. Acad Med.

[10] Hebert RS, Levine RB, Smith CG, Wright SM (2003). A systematic review of resident research curricula. Acad Med.

[11] Bierer SB, Huiju CC (2010). How to measure success: the impact of scholarly concentrations on students – a literature review. Acad Med.

[12] Ahmadi S-F, Hamid RB, Emad A (2015). Effectiveness of teaching evidence-based medicine to undergraduate medical students: A BEME systematic review. Med Teach.

[13] Maggio LA, Tannery NH, Chen HC, ten Cate O, O'Brien B (2013). Evidence-based medicine training in undergraduate medical education: a review and critique of the literature published 2006–2011. Acad Med.

[14] Willison J, O'Regan K (2007). Commonly known, commonly not known, totally unknown: a framework for students becoming researchers. High Educ Res Develop.

[15] Kern DE, Thomas PA (2009). Curriculum Development for Medical Education.

[16] Hausman JJ (1974). Mapping as an approach to curriculum planning. Curriculum Theory Net.

[17] Harden RM (2001). AMEE Guide No. 21: Curriculum mapping: a tool for transparent and authentic teaching and learning. Med Teach.

[18] Denny JC, Smithers JD, Armstrong B, Spickard A (2005). 'Where do we teach what?' Finding broad concepts in the medical school curriculum. J Gen Intern Med.

[19] Green ML (2001). Identifying, appraising, and implementing medical education curricula: a guide for medical educators. Ann Intern Med.

[20] Lammerding-Köppel M, Biller S, Jünger J, Obertacke U (2013). MERLIN-Projekt: Kompetenzorientiert lernen, lehren und prüfen in der Medizin Baden-Württemberg.

[21] Mayring P (2015). Qualitative Inhaltsanalyse. Grundlagen und Techniken.

[22] Hautz SC, Hautz WE, Keller N, Feufel MA, Spies C (2015). Die Gelehrten-Rolle im Nationalen Kompetenzbasierten Lernzielkatalog Medizin (NKLM) im Vergleich zu anderen internationalen Rahmenwerken. GMS Ger Med Sci.

[23] Frank JR (2005). The CanMEDS 2005 physician competency framework. Better standards. Better physicians. Better care.

[24] Metz JC, Verbeek-Weel AM, Huisjes HJ (2001). Blueprint 2001: training of doctors in the Netherlands. Adjusted objectives of undergraduate medical education in the Netherlands.

[25] Laidlaw A, Aiton J, Struthers J, Guild S (2012). Developing research skills in medical students: AMEE Guide No. 69. Med Teach.

[26] Loos S, Sander M, Albrecht M (2014). Systematische Situationsanalyse zum wissenschaftlichen Nachwuchs in der klinischen Forschung.

[27] Khan KS, Coomarasamy A (2006). A hierarchy of effective teaching and learning to acquire competence in evidenced-based medicine. BMC Med Educ.

[28] Ilic D, Maloney S (2014). Methods of teaching medical trainees evidence-based medicine: a systematic review. Med Educ.

[29] Harden RM (1999). What is a spinal curriculum?. Med Teach.

[30] Biggs J (2006). Enhancing teaching through constructive alignment. High Educ.

[31] Biggs J (2009). What the student does: teaching for enhanced learning. High Educ Res Develop.

[32] Treleaven L, Voola R (2008). Integrating the Development of Graduate Attributes Through Constructive Alignment. J Market Educ.

[33] Eckel J, Rolletschek A, Schüttpelz-Brauns K, Miethke T, Fritz HM (2015). Stärkung wissenschaftsorientierter Kompetenzen im Mannheimer Reformierten Curriculum für Medizin (MaReCuM) – Entwicklung einer Toolbox zur wissenschaftsorientierten Lehre. http://dx.doi.org/10.3205/15gma216.

